# Cutaneous tuberculosis as metastatic tuberculous abscess

**DOI:** 10.1590/S1806-37132015000004388

**Published:** 2015

**Authors:** Cecília Pacheco, Eloísa Silva, José Miranda, Raquel Duarte

**Affiliations:** 1 Pulmonology. Hospital de Braga, Pulmonology Department, Braga, Portugal, Department, Hospital de Braga, Braga, Portugal; 2 Pulmonology. Centro Hospitalar Tondela-Viseu, Department, Viseu, Portugal, Pulmonology Department, Centro Hospitalar Tondela-Viseu, Viseu, Portugal; 3 Cardiothoracic Surgery. Centro Hospitalar de Vila Nova de Gaia, Department, Vila Nova de Gaia, Portugal, Cardiothoracic Surgery Department, Centro Hospitalar de Vila Nova de Gaia/Espinho Hospital EPE, Vila Nova de Gaia, Portugal; 4 Pulmonology. Centro Hospitalar de Vila Nova de Gaia, Department, Vila Nova de Gaia, Portugal, Pulmonology Department, Centro Hospitalar de Vila Nova de Gaia/Espinho, EPE, Vila Nova de Gaia, Portugal; Referral Centre for Multidrug-resistant tuberculosis in the Northern Region of Portugal, Chest Disease Centre of Vila Nova de Gaia, Vila Nova de Gaia, Portugal; Institute of Public Health at University of Porto, Porto, Portugal

## To the Editor:

Cutaneous tuberculosis (CTB) continues to be one of the most difficult diagnoses to make because of the wide variations in its clinical appearance, histopathology, immunology and treatment response.^(^ 1 ^,^ 2 ^)^ The incidence of this disease has increased in the 21st century, due to a high incidence of HIV infection and multidrug-resistant pulmonary tuberculosis.^(^ 3 ^,^ 4 ^)^

Although CTB accounts for only 1.5% of all cases of extrapulmonary tuberculosis and 0.15% of all cases of skin disease, given the high prevalence of tuberculosis in many countries, these numbers are significant.^(^ 1 ^,^ 2 ^)^ *Mycobacterium tuberculosis*, *M. bovis*, and the BCG vaccine can all cause CTB.^(^ 5 ^)^

In most cases, tuberculosis is transmitted via the airborne route, and skin manifestations are a result of hematogenous spread or direct extension from a focus of infection. However, primary inoculation can occur through direct introduction of the mycobacteria into the skin or mucosa of a susceptible individual by trauma or injury. The risk increases in the presence of HIV infection, intravenous drug abuse, diabetes mellitus, immunosuppressive therapy, malignancy, end-stage renal disease, or infancy.^(^ 5 ^,^ 6 ^)^ Albeit a rare sign, CTB should be considered in the differential diagnosis of skin lesions, especially in individuals with a history of tuberculosis.

**Figure 1 -:**
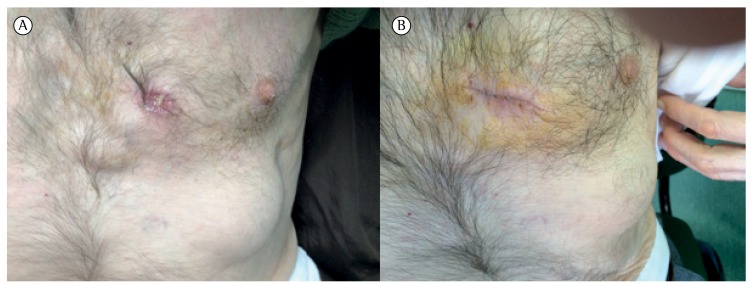
Before and after treatment (photographs on the left and right, respectively).

A 68-year-old male presented with a six-month history of weight loss and asthenia. He was a retired factory worker and former smoker, with a history of pulmonary tuberculosis in his youth (two distinct episodes, 20 years apart, the treatment regimens employed in those episodes being unknown), schizophrenia, osteoarticular pathology, and reflux esophagitis. The patient also presented with two anterior thoracic skin swellings (22 × 60 cm and 80 × 30 cm, respectively) that were painful on palpation, with an elastic consistency and without local warmth on the overlying skin (Figure 1). He reported that the swellings had first appeared one month earlier. He reported no fever or respiratory complaints. No lymph nodes were detected. A CT scan of the chest showed two liquid collections in the anterior chest wall, with a dystrophic aspect, together with thickening of the costal arch and the adjacent costal cartilage.

Diagnoses such as staphylococcal abscess, mixed bacterial infection, nocardiosis, atypical mycobacterial infections, and deep fungal infections were considered. A biopsy of one of the swellings showed granulation tissue with lymphocytes, plasma cells, and histiocytes, with suppurative areas and a fistulous tract. In sputum smears, staining for AFB was negative, although a PCR of a sputum sample was positive for* M. tuberculosis*. Serology for HIV was negative. The patient was referred to a center for the treatment of thoracic diseases, for evaluation and treatment.

Another CT scan of the pulmonary parenchyma revealed some fibrotic changes in both lung apices. Sputum smear staining for AFB was again negative, although a culture of the biopsy sample was positive for* M. tuberculosis*. Drug susceptibility testing showed that the strain was susceptible to isoniazid and rifampin, as well as to all of the first-line antituberculosis drugs tested. 

The patient was started on four antituberculosis drugs, at doses adjusted for body weight-isoniazid (300 mg/day); rifampin (600 mg/day); pyrazinamide (1,500 mg/day); and ethambutol (1,000 mg/day). Psychiatric evaluation was requested in order to adjust the regimen of treatment to take the schizophrenia into account. After two months of treatment, the tuberculosis treatment regimen was reduced to two drugs-isoniazid (300 mg/day) and rifampin (600 mg/day). At this writing, the patient has completed six months of treatment without major side effects and the skin lesions have improved (Figure 1).

The description of CTB includes dermatological manifestations of tuberculosis involving the skin, and early classifications of this disease were based on lesion morphology.^(^ 1 ^,^ 3 ^,^ 5 ^)^ In patents with CTB, skin lesions are characterized by granulomatous inflammation, varying degrees of necrosis, and varying degrees of vasculitis; *M. tuberculosis* is identified by special staining, culture, or PCR.^(^ 3 ^,^ 5 ^,^ 6 ^)^

**Chart 1 -:**
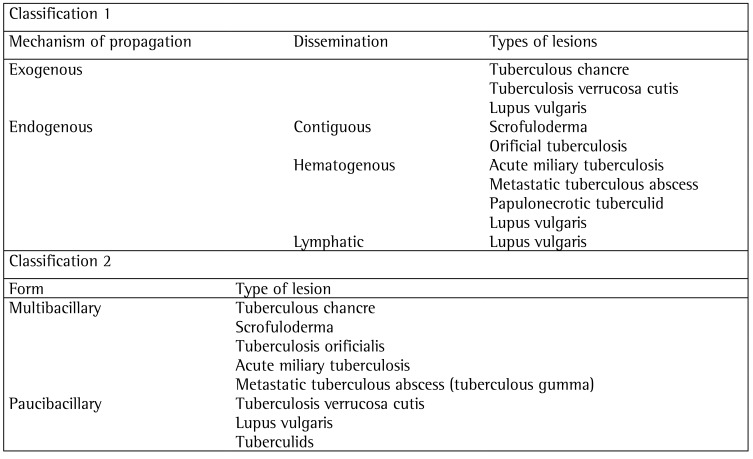
Classifications for cutaneous tuberculosis.

Despite being clinically similar, individual CTB lesions can present with different development, progression, and prognosis. On the basis of that knowledge, Tappeiner & Wolff proposed the most widely accepted CTB classification system, which is based on the mechanism of propagation-exogenous versus endogenous dissemination.^(^ 3 ^,^ 5 ^)^ Exogenous inoculation occurs after the direct inoculation of *M. tuberculosis* into the skin of a person who is susceptible to infection. Endogenous infection occurs in patients who were previously infected. Exogenous transmission is significantly less common.^(^ 5 ^)^ After that first classification, other authors introduced the concept of bacterial load, which is used in order to differentiate between the multibacillary form (in which mycobacteria are easily identified on histological examination) and the paucibacillary form (in which isolation of mycobacteria in culture is rare).^(^ 1 ^,^ 3 ^,^ 5 ^)^ Chart 1 summarizes those two classification systems.

Here, we have reported the case of a patient ultimately diagnosed with metastatic tuberculous abscess, also known as tuberculous gumma, which is normally due to hematogenous spread of mycobacteria that remain latent until, for some reason (e.g., immunosuppression or malnutrition), the infection manifests itself, malnutrition being the probable trigger in our case. This form of CTB is characterized by non-tender and fluctuant subcutaneous abscesses, appearing as single or multiple lesions on the trunk, extremities, or head, which often invade the skin and break down. 

Although making a clinical diagnosis of CTB is not always easy, it should be considered in all cases of chronic skin lesions, mainly in HIV-infected patients and in patients with history of pulmonary tuberculosis.^(^ 6 ^)^ The differential diagnosis includes staphylococcal abscess, other mixed bacterial infections, sporotrichosis, nocardiosis, chromomycosis, leishmaniasis, atypical mycobacterial infections, deep fungal infections, syphilitic gumma, leprosy, and all forms of panniculitis.^(^ 2 ^)^

Supporting evidence for the clinical presentation includes epidemiological data, history of tuberculosis or contact with a tuberculosis patient, and histology (skin biopsy in most cases). Histology must include sputum smear testing for AFB, PCR, and culture for *M. tuberculosis*. ^(^ 1 ^-^ 3 ^,^ 5 ^,^ 6 ^)^ Isolating *M. tuberculosis* in culture is the only way to make a definitive diagnosis.

Tuberculous gumma is a multibacillary form of CTB that can occur without any underlying source of tuberculosis. Histology of CTB lesions reveals massive necrosis and abscess formation. Staining for AFB usually shows large quantities of mycobacteria.^(^ 1 ^,^ 3 ^)^

The antituberculosis drug regimens used in the treatment of pulmonary tuberculosis are adequate for treating CTB, because the bacillary load in CTB is usually much smaller than that occurring in pulmonary tuberculosis.^(^ 3 ^,^ 7 ^,^ 8 ^)^ This include regimens of directly observed therapy, and the standard treatment regimen involves two months of quadruple therapy (isoniazid, rifampin, pyrazinamide, and ethambutol) followed by four months of double therapy (isoniazid and rifampin).^(^ 1 ^)^

It is extremely important to determine the past history of tuberculosis, given that patients with such a history are more likely to be infected with a strain of mycobacterium that is resistant to antituberculosis drugs. In the case presented here, a culture of the biopsy specimen was positive for *M. tuberculosis*, and drug susceptibility testing showed susceptibility to all of the first-line drugs tested. Surgical excision is sometimes necessary, not only as a diagnostic method, but also as an adjunct to pharmacological therapy.^(^ 4 ^)^ A clinical response should be expected between weeks 4 and 6 of treatment,^(^ 1 ^)^ as was observed in our case.

Although uncommon, CTB should be kept in mind in the differential diagnosis of skin lesions.
